# Confirmation of previously identified plasma microRNA ratios for breast cancer detection in a nested case‐control study within a screening setting

**DOI:** 10.1002/ctm2.70068

**Published:** 2024-11-15

**Authors:** Emir Sehovic, Ilaria Gregnanin, Maurizia Mello‐Grand, Paola Ostano, Viviana Vergini, Andrea Ortale, Angela Amoruso, Elisabetta Favettini, Nereo Segnan, Giovanna Chiorino, Livia Giordano, Elisabetta Petracci

**Affiliations:** ^1^ Laboratory of Cancer Genomics Fondazione Edo ed Elvo Tempia Biella Biella Italy; ^2^ SSD Epidemiologia Screening Centro di Riferimento per l'Epidemiologia e la Prevenzione Oncologica in Piemonte Torino Torino Italy; ^3^ Department of Diagnostic Radiology Ospedale degli Infermi Ponderano Biella Italy; ^4^ Unit of Biostatistics and Clinical Trials Istituto Romagnolo per lo Studio dei Tumori “Dino Amadori” – IRST S.r.l. Istituto di Ricovero e Cura a Carattere Scientifico Meldola Forlì‐Cesena Italy

Dear Editor,

Circulating cell‐free microRNAs (miRNAs) were rarely explored as biomarkers for early detection of breast cancer (BC) within a screening setting or in prospectively sampled cohorts.^1^ In this study, we confirmed the discriminatory ability of a combination of novel and reliable circulating miRNA‐ratio biomarkers, with and without nonmolecular predictors, that could be used for BC early detection in the context of mammographic screening using a standard, affordable, noninvasive and reproducible technique such as quantitative Reverse Transcription Polymerase Chain Reaction (RT‐qPCR). The models were built on a discovery case‐control set (*n* = 131) nested within a large mammographic screening cohort,^2^ where more than 14 000 out of 26 640 enrolled women filled an extensive questionnaire on lifestyle habits, hormonal and reproductive history and familiarity for BC, underwent anthropometric measurements and blood sampling. A model with candidate predictors was obtained through penalised logistic regression, and the selected variables were seven plasma miRNA‐ratios, breast density, lifestyle score, menopausal status (MS), body mass index (BMI) and their interaction (BMI × MS). Area under the receiver operating characteristic curve (ROC AUC) of .79 for the complete model and of .73 for the miRNA‐only model were obtained.^3^ Here, we applied the two models to a new set of women (validation set, *n* = 159) nested within the same cohort, including cases diagnosed up to four years after blood collection. Table 1 shows the main characteristics of the sample, with a similar distribution of factors between cases and controls except for the number of previous breast biopsies, breastfeeding and waist circumference. The flowchart of the validation study is visualised in Figure 1 and the methods are detailed in the Supplementary Information. Investigating associations between sample characteristics and studied miRNAs, we found weak correlations between BMI and three miRNA‐ratios and between WCRF lifestyle score and one miRNA‐ratio (Figure S1).

**TABLE 1:** Demographic, family, reproductive and screening history, lifestyle, anthropometric measurements, education and breast density are reported for the validation set.

	Cases (*n* = 32)		Controls (*n* = 127)		Cases vs. controls	
	*N*	%	*N*	%	OR (95% CI)	*P*
**Age at enrolment (years)**						
Mean ± SD	64.70 ± 6.33		63.11 ± 5.89		1.04 (.98–1.12)	.193
**Previous benign biopsies**						
0	24	75	118	92.91	1 (Ref)	
≥ 1	6	18.75	9	7.09	3.28 (1.02–9.98)	.038
Missing	2	6.25	0	–		
**Education**						
Low	13	40.63	55	45.08	1 (Ref)	
Medium	12	37.50	55	45.08	.92 (.38–2.21)	.857
High	2	6.25	12	9.84	.71 (.10–3.02)	.671
Missing	5	15.63	5	–		
**Nr. of first‐degree relatives with BC**						
0	25	78.13	106	83.46	1 (Ref)	
≥ 1	7	21.88	21	16.54	1.41 (.51–3.57)	.480
Missing	0	0	0	–		
**Age at menarche (years)**						
≤ 11	7	21.88	27	21.26	1 (Ref)	
12–13	16	50	66	51.97	1.02 (.34–3.19)	.971
≥ 14	9	28.13	34	26.77	.94 (.36–2.66)	.895
Missing	0	0	0	–		
**Age at first full pregnancy (years)**						
Nulliparous	12	37.50	25	19.69	1.82 (.69–4.95)	.229
≥ 19	2	6.25	12	9.45	.63 (.09–2.85)	.588
20–24	10	31.25	38	29.92	1 (Ref)	
25–29	3	9.38	29	22.83	.39 (.08–1.42)	.184
≥ 30	5	15.63	23	18.11	.83 (.23–2.64)	.753
Missing	0	0	0	–		
**Contraceptive use**						
None OR < 1 year	27	84.38	100	80.65	1 (Ref)	
1–4 years	0	0	0	–		
≥ 5 years	3	9.38	24	19.35	.46 (.10–1.46)	.236
Missing	2	6.25	3	–		
**Breastfeeding**						
Nulliparous OR no breastf. OR breastf. < 6 months	28	87.50	78	61.42	1 (Ref)	
≥ 6 months	4	12.50	49	38.58	.23 (.06–.62)	.009
Missing	0	0	0	–		
**Menopausal status**						
Not in menopause	8	25	22	17.32	1 (Ref)	
Menopause	24	75	105	82.68	.63 (.26–1.66)	.324
Missing	0	0	0	–		
**HRT**						
Not in menopause	8	25	22	17.32	1 (Ref)	
No HRT use OR HRT use < 1 year	19	59.38	93	73.23	.56 (.22–1.51)	.233
≥ 1 year	3	9.38	12	9.45	.69 (.13–2.90)	.625
Missing	2	6.25	0	–		
**Measured BMI (kg/m^2^)**						
mean ± SD	26.3 ± 4.31		25.06 ± 4.88		1.05 (.97–1.14)	.194
**Waist circumference (cm)**						
Mean ± SD	89.8 ± 12.1		84.82 ± 12.35		1.03 (1.00–1.06)	.047
**Level of occupational physical activity at age 30–39 years**						
Exclusively/mainly sitting	10	31.25	33	25.98	1 (Ref)	
Standing or average	13	40.63	57	44.88	.75 (.30–1.94)	.549
Heavy or very heavy	7	21.88	37	29.13	.62 (.21–1.81)	.390
Missing	2	6.25	0	–		
**Level of leisure time physical activity at 30–39 years**						
< 2 h/week	16	50	67	52.76	1 (Ref)	
≥ 2 h/week	14	43.75	60	47.24	.98 (.44–2.17)	.955
Missing	2	6.25	0	–		
**Alcohol habit**						
Never drinker or ex drinker	6	18.75	39	30.71	1 (Ref)	
Drinker, also occasionally	24	75	88	69.29	1.77 (.71–5.09)	.248
Missing	2	6.25	0	–		
**Smoking habit**						
Never smoker	17	53.13	77	60.63	1 (Ref)	
Ex‐smoker	6	18.75	26	20.47	1.05 (.35–2.82)	.933
Occasionally/smoker	9	28.13	24	18.90	1.7 (.65–4.25)	.264
Missing	0	0	0	–		
**Tabar classification** ^*^						
1	4	12.50	50	39.37	1 (Ref)	
2	14	43.75	63	49.61	2.78 (.93–10.27)	.087
3	12	37.50	9	7.09	16.67 (4.72–71.36)	<.001
4 or 5	2	6.25	5	3.94	5 (.59–33.66)	.102
Missing	0	0	0	–		
**WCRF lifestyle score**						
Mean ± SD	5.08 ± 1.34		5.37 ± 1.19		.83 (.61–1.13)	.229

*Note*: Absolute frequencies (*N*) and percentages (%) are reported for categorical variables, whereas mean ± standard deviation (SD) are reported for continuous variables. Odds ratios (OR) and *p*‐values (*P*) of univariable logistic regression analysis are also reported.

* When considering Tabar breast density classifications as linear, an OR of 2.57 [1.58–4.36] was obtained with a *p*‐value of .0002.

breastf = breastfeeding; HRT = hormone replacement therapy; WCRF = world cancer research fund.

**FIGURE 1:**
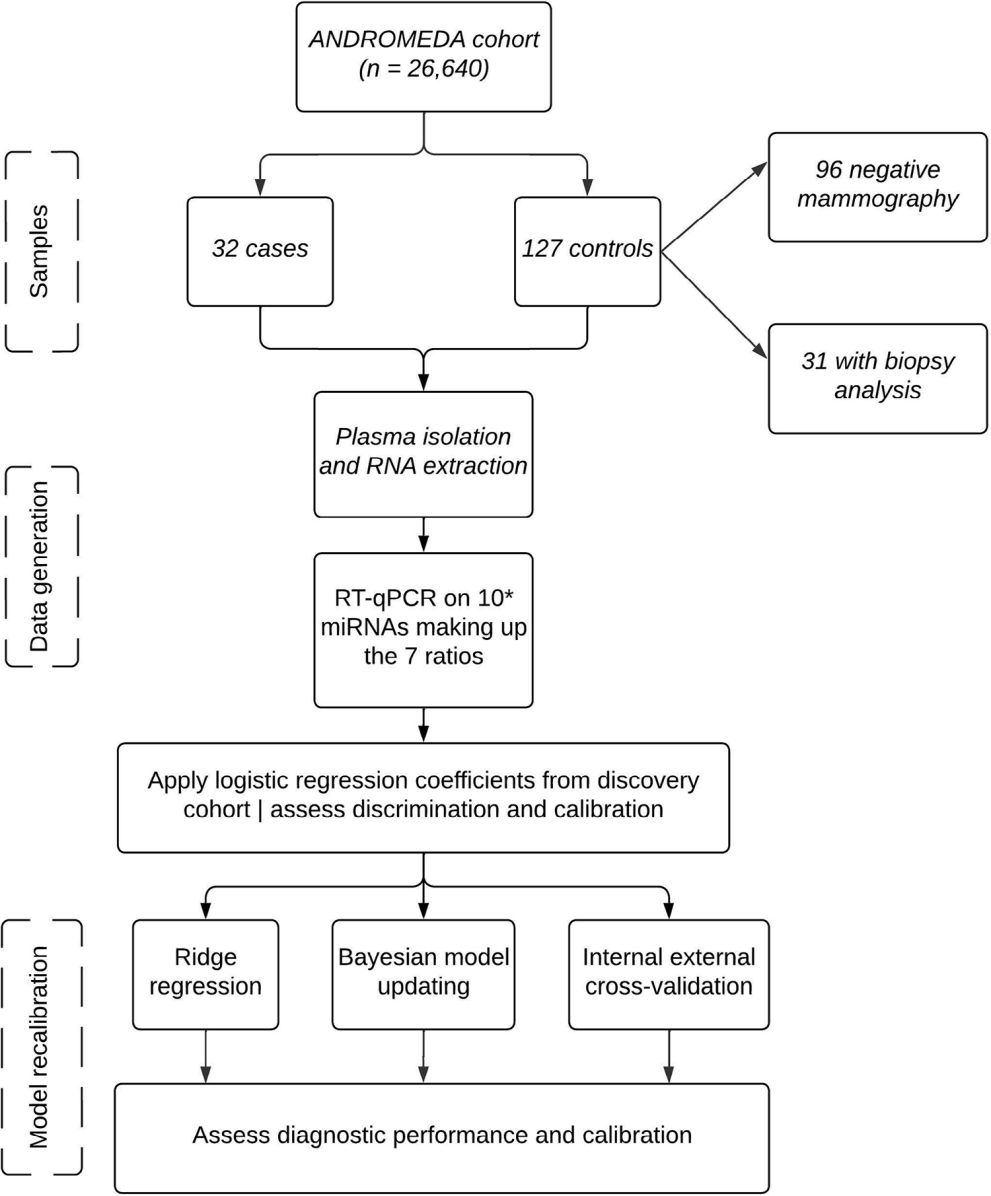
Flowchart of the pipeline in which we assessed the discrimination power and calibration of the model on miRNA ratios and nonmolecular variables. *let‐7b‐5p was replaced by let‐7a‐5p in the ratios, due to a high correlation (*ρ* = .96) of Cts between the two miRNAs within the discovery set and as their mature sequences are almost identical (two nucleotides are different).

The cancer characteristics in the discovery and validation sets were similar. However, in the discovery set the diagnosis occurred earlier relative to blood sampling (average 3 months vs. average 25 months), and the proportion of ki‐67 positive tumours was lower (23 .5% vs. 82%) (Table S1). Moreover, unlike the discovery set, the validation set included 31 controls that underwent second‐level investigation after a suspicious mammogram but then had a negative biopsy. The variables selected in the discovery model were comparable between the two control subgroups (Table S2). Two miRNA‐ratios (miR‐199a‐3p/let‐7a‐5p and miR‐26b‐5p/miR‐142‐5p) were associated with ER status, with *p*‐values of .049 and .027, respectively. Additionally, miR‐93‐5p/miR‐19b‐3p was associated with PgR status (*p* = .036) and let‐7b‐5p/miR‐19b‐3p with Tabar's classification of breast density (*p* = .025) (Figure S2).

We applied the coefficients of nonmolecular variables and miRNA‐ratios (obtained in the discovery set) to the validation set, yielding subpar discriminatory ability (Figure S3A), with ROC AUC = .63 (95% CI: .53–.74) and Brier score of .43. To assess the model calibration, a calibration curve was computed, and its intercept and slope were analysed. The predicted probabilities were miscalibrated, with a substantial overestimation of BC risk (Figure S3B), probably due to the differences between the two sets. The closed testing procedure indicated that the most appropriate model updating method was the re‐estimation of the intercept and coefficients. After model recalibration using penalised ridge logistic regression, we obtained an ROC AUC of .87 (.81–.93) (Figure 2A), a Brier score of .11 and robust estimates after bootstrapping (Figure 2B). The sensitivity and specificity at Youden's cut‐off (.17) were .97 and .70, respectively, and the calibration of the predicted probabilities was improved (Figure 2C). Using univariate logistic regression, we also investigated the seven miRNA‐ratios in other publicly available circulating miRNA datasets, and despite the technological and population differences, subsets of the seven miRNA‐ratios were associated with BC (Table S3).

**FIGURE 2:**
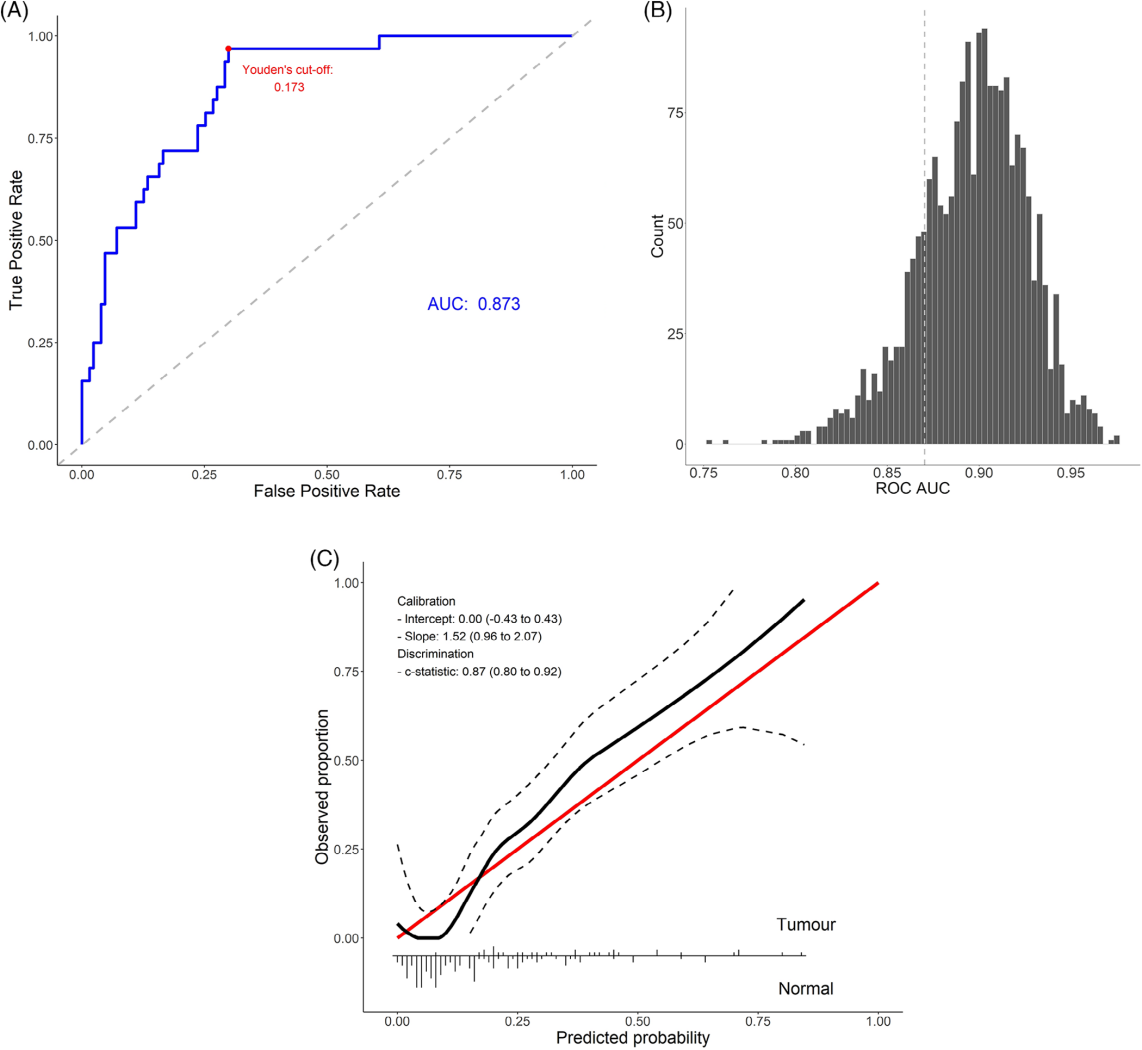
ROC AUC (A) based on the ridge regression on the model with all predictors. Within the ROC curve, the Youden's cut‐off and AUC are reported. (B) Histogram of bootstrapped (2000 sampling iterations) ROC AUC based on the ridge regression. The vertical dashed line represents the ROC AUC obtained in the ridge logistic regression model on the original validation sample. (C) Calibration plot of the ridge regression model on all predictors where the intercept and slope of the calibration curve and the *c* statistic are shown.

Furthermore, we merged the individual patient data of the discovery and validation sets and performed the internal external cross‐validation (IECV). Using this approach, we created a new discriminatory model and accounted for the two datasets. The IECV model on all merged predictors had a relatively large heterogeneity on the meta‐analysed Brier score (tau^2^ = .054). Therefore, the model on the combined dataset, based on all selected predictors, was not more informative than the models obtained from individual sets. A notable limitation of the IECV method in this study is the inclusion of only two cohorts, resulting in relatively unstable meta‐analysis estimates. Nevertheless, we utilised the IECV method to create a model with the most generalisable predictors, obtaining a relatively low heterogeneity on the meta‐analysed Brier score (tau^2^ = .001). This model included five predictors (miR‐21‐5p/miR‐23a‐3p, miR‐199a‐3p/let‐7a‐5p, MS, breast density and BMI), and had well‐calibrated predicted probabilities (Figure S4) with a Brier score of .17 and an ROC AUC of .79 (.73–.85). The two miRNA‐ratios were the predictors with coefficients of highest magnitude in the updated model (Table 2), suggesting a stronger diagnostic potential. A model combining three circulating small RNAs including miR‐23a‐3p and a miR‐21‐5p isoform was shown to discriminate stage 0 BCs from controls (ROC AUC of .92), although the study was not conducted within a screening set nor with prospective enrolment.^4^ Additionally, among the analysed datasets in Table S3, the most concordant miRNA‐ratios (based on the direction of the relationship as summarised by the odds ratio) were the two with highest generalisability according to the IECV.

**TABLE 2:** Re‐calibrated coefficients in the validation set of the predictors included in the two models.

	Complete model	miRNA ratio‐only model
Intercept	−9.58	−5.02
miR‐199a‐3p_let‐7a‐5p	1.09	.61
miR‐26b‐5p_miR‐142‐5p	.05	.10
let‐7b‐5p_miR‐19b‐3p	–.47	–.36
miR‐101‐3p_miR‐19b‐3p	.22	.21
miR‐93‐5p_miR‐19b‐3p	.15	–.46
let‐7a‐5p_miR‐22‐3p	.2	.12
miR‐21‐5p_miR‐23a‐3p	.98	
Menopausal status	–.24	
BMI	.59	
BMI × Menopause	–.16	
WCRF lifestyle score	–.11	
Breast density	.96	

Analogous results were obtained for the miRNA‐ratio‐only model, with relatively poor performance after initial application and miscalibrated predictive probabilities (Figure S5). After model updating, we obtained an ROC AUC of .77 (.69–.85), with a substantial improvement of the predicted probabilities (Brier score = .14) (Figure S6).

We also assessed the performance of the identified plasma miRNA‐ratios in 103 paired samples from breast cancer TCGA dataset, and all but let‐7a‐5p/miR‐22‐3p were significantly deregulated in breast tumours relative to normal adjacent tissues (Table S4). Hence, it is plausible that these miRNA‐ratios have a role in BC onset or progression.

Most of the thus‐far published results of diagnostic cell free circulating miRNAs in the context of BC have focused on miRNA levels in patients already diagnosed with BC and lack methodological standardisation.^1^, ^5^ Thus, it remains unclear whether these biomarkers can be used for risk stratification or if they are merely a consequence of cancer progression, and whether they are generalisable. The key strengths of this study are the prospective sampling before any kind of treatment or diagnosis and the usage of miRNA‐ratios for which RT‐qPCR normalisers are not necessary.^6^

In conclusion, we validated the discriminatory ability, in a screening setting, of candidate miRNA‐ratio biomarkers that could easily be applied in the clinics and identified which of them are most generalisable. Although building a model on a larger dataset with more BC cases is needed, we highlighted the potential of plasma miRNAs, alone or combined with lifestyle and individual characteristics, for BC precision screening (Graphical abstract).

## AUTHOR CONTRIBUTIONS

ES: statistical data analysis, microRNA assessment, manuscript conceptualisation, writing and revision; IG: sample collection, processing, storing, microRNA assessment, manuscript revision; MMG: sample collection, processing, storing; PO: data analysis and data upload to Zenodo; VV: database management and case‐control selection; AO: database generation and data elaboration; AA: mammogram analysis and breast density evaluation; EF: mammogram analysis; NS: funding and manuscript revision; GC: funding, supervision of experimental and analysis work, manuscript conceptualisation, writing and revision; LG: supervision of clinical data analysis, manuscript revision; EP: statistical data analysis supervision, manuscript conceptualisation, writing and revision.

## FUNDING INFORMATION

The project was funded by an investigator grant from the Italian Association for Cancer Research (AIRC IG 2014 Ref No 15374) to NS and LG, by the European Union's Horizon 2020 Research and Innovation Programme, Marie Skłodowska‐Curie (Grant Number 859860) to ES and GC and by the 106562/RF 2023.1638 grant from Fondazione CRT to IG, MM‐G and GC.

## ETHICS STATEMENT

Ethical approval was obtained from the Ethics Committee of each participating centre (Ethical and deontological institutional review board of the A.O.U Città della Salute e della Scienza of Turin, with the protocol number 78326 on 11.07.2013 and Ethical Committee of Novara with the protocol number 248/CE and study number CE 27/15).

## Supporting information



Supporting Information

Supporting Information

Supporting Information

Supporting Information

Supporting Information

Supporting Information
