# Supplementary material for: Confirmation of previously identified plasma microRNA ratios for breast cancer detection in a nested case‐control study within a screening setting
Source: Clin Transl Med. 2024 Nov 15;14(11):e70068. doi: 10.1002/ctm2.70068 (PMC11567874; doi:10.1002/ctm2.70068)
Supplement: Supplementary file 1 — Supporting Information [file CTM2-14-e70068-s004.docx]

# Methods

## Study population

To investigate the performance of the previously identified biomarkers, a case-control study nested into the ANDROMEDA cohort, was conducted. The ANDROMEDA cohort (NTC02618538) was described in more detail elsewhere^1,2^. Ethical approval was obtained from the Ethics Committee of each participating centre (Ethical and deontological institutional review board of the A.O.U Città della Salute e della Scienza of Turin, with the protocol number 78326 on 11.07.2013 and Ethical Committee of Novara with the protocol number 248/CE and study number CE 27/15). For the present study, only the patients enrolled in the Biella centre were considered. All histologically confirmed breast cancer (BC) cases not included in the previous case-control study, were extracted together with a random sample of controls (not previously used) with a ratio of 1:3. Moreover, an additional pool of controls was considered, that is, women with a suspicious mammography, who underwent a biopsy for a histological examination of breast tissue but were in the end confirmed to be negative for BC. Controls were selected among those with a date of enrolment into the cohort similar to that of the cases (± 1 year).

All variables used in the present study, including the World Cancer Research Fund (WCRF) lifestyle score as suggested by Romaguera and colleagues^3^ and mammographic breast tissue composition classification according to Tabar, were coded in exactly the same way as in our previous case-control study^1^.

## Blood collection, RNA extraction and RT-qPCR analysis

Blood sampling and plasma isolation as well as the RNA extraction and RT-qPCR analysis have already been thoroughly explained in our previous work on the ANDROMEDA cohort^1,4^. The only difference in miRNA assaying using RT-qPCR was in that we used duplicate Ct measurements for each sample instead of triplicate.

A total of seven miRNA ratios were selected in the discovery study and the miRNAs making up the ratios (miR-199a-3p_let-7a-5p, miR-26b-5p_miR-142-5p, let-7b-5p_miR-19b-3p, miR-101-3p_miR-19b-3p, miR-93-5p_miR-19b-3p, let-7a-5p_miR-22-3p and miR-21-5p_miR-23a-3p) were assayed in this case-control sample using qRT-PCR. Importantly, to enable overall much more cost-efficient RT-qPCR runs, let-7b-5p was replaced by let-7a-5p in the ratio it was a part of since their threshold cycles (Cts) were found to be highly correlated (ρ = 0.96) and their mature sequences are very similar, with only two purine nucleotides being different.

## Statistical analysis

The statistical analyses were performed in the R software (version 4.4.0)^5^.

The LASSO logistic regression coefficients of the variables included in the two models reported in our previously published case-control study (discovery set)^1^, were applied to the present case-control study (validation set) using the sigmoid logistic regression function to obtain the BC predicted probabilities. In the validation set, as previously done for the discovery set, the RT-qPCR miRNA ratios were computed by average Ct(Y) − average Ct(X), where Y is the denominator and X is the numerator of the original ratios.

Model performances were assessed using the ROC AUC and the Brier score of the predictions as well as measures of calibration. To evaluate calibration, we computed a logistic calibration curve, which was obtained by fitting a logistic regression model where the dependent variable was the case-control status and the independent variable was the log odds of the predicted probabilities. This was done using the “val.prob.ci.2” function in the *CalibrationCurves* package^6–8^. We then investigated the calibration intercept (‘calibration-in-the-large’) and the calibration slope, as explained in the vignette of the package. Briefly, the slope of the calibration curve measures the overfitting (slope < 1) and underfitting (slope > 1) of the model, while the calibration intercept measures whether the predicted probabilities are overestimated (intercept < 0) or underestimated (intercept > 0) on average.

We used the so-called closed-testing procedure to select the optimal way of model updating^9^. This procedure is based on a series of likelihood ratio tests, comparing different ways of model updating (i.e., complete model revision, intercept and slope recalibration or only the intercept calibration) and the original model. We selected a p-value threshold of 0.05 to assess that a model updating method is significantly better than the original model or the other model updating method. Details on how the likelihood ratio test is performed is described in the original article^9^. Within the same article, an R function was reported for performing the closed testing procedure and was implemented in this study. The reason for performing this procedure is to avoid increasing the Type I error by assessing all the model updating options separately. For complete model re-evaluation (including the coefficients, intercept and slope), we used the ridge regression in order to reduce some of the overoptimism and overfitting which would arise from a regular logistic regression. The function used was “cv.glmnet” from the *glmnet* package^10,11^ with alpha being set at 0. The discriminatory ability and calibration of the resulting model, as well as all other re-calibrated models, were assessed using apparent validation, i.e. application of the model on the dataset where it was built. Further, to account for overfitting and gain additional insight of the performance range on the re-evaluated model, we performed an ordinary bootstrap resampling (B = 2000), using the “boot” function from the *boot* package^12,13^, on which the model calibration using ridge regression was performed and ROC AUC calculated based on apparent validation. From the resulting distribution of ROC AUCs, a 95% confidence interval was obtained using the “boot.ci” function.

We employed the Bayesian model updating as an alternative model calibration method, used as a comparison to the frequentist ridge regression. In the Bayesian model updating, the coefficients of the discovery were used as means of prior probabilities and a constant of log(4)/2 was used for the standard deviation of the priors^14,15^. Bayesian model updating was performed using the *brms* package^16–18^.

Finally, we used the internal-external cross-validation (IECV) method to merge the discovery and validation sets to create a new model while accounting for the sets or to identify generalisable predictors. IECV merges the individual participant data and then trains the model on K-1 sets (in this case case-control sets) and tests it on the remaining one^19^. The model coefficients obtained in the K-1 sets were combined using standard meta-analysis techniques. A random effect meta-analysis is used to summarise estimates of prediction model performance (Brier score), and the between-study variance (tau^2^) of this estimate represents the heterogeneity between the merged sets. Importantly, in case of large heterogeneity the model on merged sets is less informative than models on the separate sets. Moreover, the IECV method can also start from an intercept-only model and iteratively add predictors until the heterogeneity is optimised for the cohorts. We exploited this method to identify the most generalisable predictors between our two datasets. The IECV was performed using the *metamisc* package^20^ and we used the restricted maximum likelihood (REML) random effect meta-analysis within the “metapred” function.

## Circulating miRNA expression in other studies

We investigated the expression profiles of the seven miRNA ratios in other publicly available circulating miRNA datasets. We selected datasets of studies that collected blood samples and performed subsequent miRNA analysis of BC patients and healthy controls. Within the selection we included studies using any type of platform for data generation. Importantly, the six analysed datasets were heterogeneous and different from our sample in many aspects such as population studied, technology used, and sample size. Another important distinction to our study is the fact that all six datasets performed retrospective sampling.

For microarray miRNA expression studies, we downloaded the normalised matrices, while for NGS and qRT-PCR studies we downloaded the raw expression counts and Ct values, respectively. Depending on the platform, the miRNA ratios were computed from the obtained datasets in the following way:

1. Log_2_ intensity (miRNA_x_) – log_2_ intensity (miRNA_y_) for microarray datasets
2. Ct (miRNA_y_) – Ct (miRNA_x_) for qRT-PCR datasets
3. Log_2_(count (miRNA_x_) / count (miRNA_y_)) for NGS datasets

Using the “glm” function in R, a univariate logistic regression was performed on each of the seven miRNA ratios computed from the analysed datasets. From this analysis, the odds ratio and its confidence interval, together with the p-value, were extracted. The GEO IDs and relevant characteristics of the analysed datasets can be seen in **Table M1**.

**Table M1.** Characteristics of the analysed studies which profiled circulating miRNAs in BC patients and healthy controls.

| **GEO ID (year)** | **Type of biological sample** | **Type of technology** | **Sample size** | **Population** | **Availability of relevant sample data** |
| --- | --- | --- | --- | --- | --- |
| GSE118782 (2019) | Plasma | Affymetrix Multispecies miRNA-1 Array | 30 breast cancer patients and 10 healthy controls | Unknown | Sample type, Tumour grade, ER time, BC status |
| GSE22981  (2011)^21^ | Plasma | Illumina Human v2 MicroRNA expression beadchip | 20 women with early stage (stage I and II) breast cancer and 20 matched healthy controls | African-American and Caucasian-American | No |
| GSE41526  (2012)^22^ | Plasma | Illumina Human v2 MicroRNA expression beadchip | 20 breast cancer cases, 20 screening mammography-based controls (age and race matched) | African-American and Caucasian-American | No |
| GSE41922  (2013)^23^ | Plasma | Exiqon LNA RT-PCR Human panels (1 & 2) | 32 breast cancer cases and 22 healthy controls (volunteers) | Singaporeans of Chinese ancestry | Estrogen receptor, HER2, node positivity, age, disease state |
| GSE73002  (2016)^24^ | Serum | 3D-Gene Human miRNA V20_1.0.0 | 1280 breast cancer cases, 2836 non-cancer controls | Japanese | No |
| GSE113486  (2018)^25^ | Serum | 3D-Gene Human miRNA V21_1.0.0 | 100 non-cancer controls, 40 breast cancer cases | Japanese | No |

## miRNA tissue expression

To gain additional insights on the miRNAs included in the ratios, an external public datataset was used. In particular, we downloaded the TCGA processed sequencing counts of miRNAs on BC tumour and adjacent healthy tissue (in February 2023). This was done using the “gdcRNADownload” function from the *GDCRNATools* package^26^. The project.id was set to “TCGA-BRCA” and the data.type to “miRNAs”. Metadata was then obtained and merged using “gdcParseMetadata” and “gdcRNAmerge” functions, respectively. The metastatic samples, as well as duplicate replicates from Formalin-Fixed Paraffin-Embedded (FFPE) blocks, were excluded from all analyses, for a total of 1,078 cases and 104 controls. There were 103 paired tumour and healthy tissue samples. We computed pairwise ratios to obtain the same list of ratios analysed in this study and performed a paired Wilcoxon two-sample test on the paired tumour and adjacent healthy tissues. The mean fold change for each ratio was calculated by taking the mean of the fold changes across the paired samples.

## Data availability

For the discovery and validation sets, miRNA ratios and non-molecular variables are available at the following link: <https://zenodo.org/records/11234225>, as well as data on the type of control for the validation set only. Raw threshold cycles (Ct) are available upon request. The code is publicly available in the github repository accessible through the following link: <https://github.com/esehovic/miRNA_ratio_test_andromeda>.

References

1. Chiorino, G. *et al.* Plasma microRNA ratios associated with breast cancer detection in a nested case–control study from a mammography screening cohort. *Sci Rep* **13**, 12040 (2023).

2. the Andromeda working group *et al.* The ANDROMEDA prospective cohort study: predictive value of combined criteria to tailor breast cancer screening and new opportunities from circulating markers: study protocol. *BMC Cancer* **17**, 785 (2017).

3. Romaguera, D. *et al.* Is concordance with World Cancer Research Fund/American Institute for Cancer Research guidelines for cancer prevention related to subsequent risk of cancer? Results from the EPIC study. *The American Journal of Clinical Nutrition* **96**, 150–163 (2012).

4. Mello-Grand, M. *et al.* Circulating microRNAs combined with PSA for accurate and non-invasive prostate cancer detection. *Carcinogenesis* **40**, 246–253 (2019).

5. R Core Team (2024). R: A Language and Environment for Statistical Computing. R Foundation for Statistical Computing (2024).

6. Campo, B. D. C. Towards reliable predictive analytics: a generalized calibration framework. Preprint at http://arxiv.org/abs/2309.08559 (2023).

7. Campo, B. D. C., Nieboer, D., Van Calster, B., Steyerberg, E. & Vergouwe, Y. CalibrationCurves: Calibration Performance. (2023).

8. Van Calster, B. *et al.* A calibration hierarchy for risk models was defined: from utopia to empirical data. *Journal of Clinical Epidemiology* **74**, 167–176 (2016).

9. Vergouwe, Y. *et al.* A closed testing procedure to select an appropriate method for updating prediction models: Method selection to update a prediction model. *Statist. Med.* **36**, 4529–4539 (2017).

10. Friedman, J., Hastie, T. & Tibshirani, R. Regularization Paths for Generalized Linear Models via Coordinate Descent. *J. Stat. Soft.* **33**, (2010).

11. Tay, J. K., Narasimhan, B. & Hastie, T. Elastic Net Regularization Paths for All Generalized Linear Models. *J. Stat. Soft.* **106**, (2023).

12. Davison, A. C. & Hinkley, D. V. *Bootstrap Methods and Their Application*. (Cambridge University Press, 1997). doi:10.1017/CBO9780511802843.

13. Canty, A. & Ripley, B. D. boot: Bootstrap Functions. (2024).

14. Siregar, S., Nieboer, D., Versteegh, M. I. M., Steyerberg, E. W. & Takkenberg, J. J. M. Methods for updating a risk prediction model for cardiac surgery: a statistical primer. *Interactive CardioVascular and Thoracic Surgery* **28**, 333–338 (2019).

15. Rothman, K. J., Greenland, S. & Lash, T. L. *Modern Epidemiology*. (Wolters Kluwer Health/Lippincott Williams & Wilkins, Philadelphia, 2008).

16. Bürkner, P.-C. brms: An R Package for Bayesian Multilevel Models Using Stan. *J. Stat. Soft.* **80**, (2017).

17. Bürkner, P.-C. Advanced Bayesian Multilevel Modeling with the R Package brms. *The R Journal* **10**, 395 (2018).

18. Bürkner, P.-C. Bayesian Item Response Modeling in R with brms and Stan. *J. Stat. Soft.* **100**, (2021).

19. De Jong, V. M. T., Moons, K. G. M., Eijkemans, M. J. C., Riley, R. D. & Debray, T. P. A. Developing more generalizable prediction models from pooled studies and large clustered data sets. *Statistics in Medicine* **40**, 3533–3559 (2021).

20. Debray, T. & de Jong, V. metamisc: Meta-Analysis of Diagnosis and Prognosis Research Studies. (2022).

21. Zhao, H. *et al.* A pilot study of circulating miRNAs as potential biomarkers of early stage breast cancer. *PLoS One* **5**, e13735 (2010).

22. Leidner, R. S., Li, L. & Thompson, C. L. Dampening enthusiasm for circulating microRNA in breast cancer. *PLoS One* **8**, e57841 (2013).

23. Chan, M. *et al.* Identification of circulating microRNA signatures for breast cancer detection. *Clin Cancer Res* **19**, 4477–4487 (2013).

24. Shimomura, A. *et al.* Novel combination of serum microRNA for detecting breast cancer in the early stage. *Cancer Sci* **107**, 326–334 (2016).

25. Usuba, W. *et al.* Circulating miRNA panels for specific and early detection in bladder cancer. *Cancer Sci* **110**, 408–419 (2019).

26. Ruidong Li, H. Q. GDCRNATools: an R/Bioconductor package for integrative analysis of lncRNA, mRNA, and miRNA data in GDC. Bioconductor https://doi.org/10.18129/B9.BIOC.GDCRNATOOLS (2018).
