# Supplementary material for: Confirmation of previously identified plasma microRNA ratios for breast cancer detection in a nested case‐control study within a screening setting
Source: Clin Transl Med. 2024 Nov 15;14(11):e70068. doi: 10.1002/ctm2.70068 (PMC11567874; doi:10.1002/ctm2.70068)
Supplement: Supplementary file 2 — Supporting Information [file CTM2-14-e70068-s003.docx]

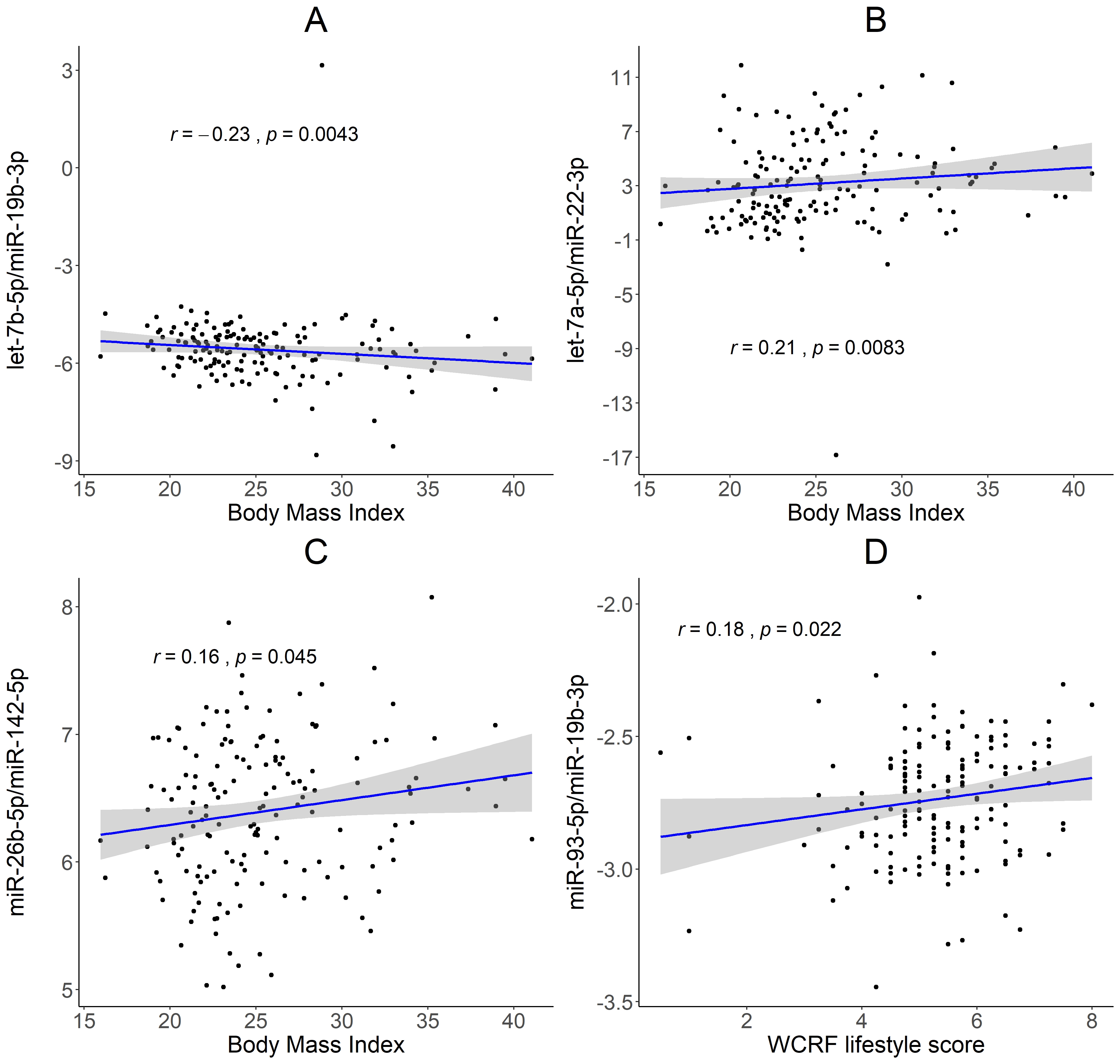


**Figure S1.** Scatter plots of miRNA-ratios associated with BMI (A-C) and WCRF lifestyle score (D). Within each plot, a regression line with its confidence interval as well as the Spearman correlation coefficient and p-value are shown.


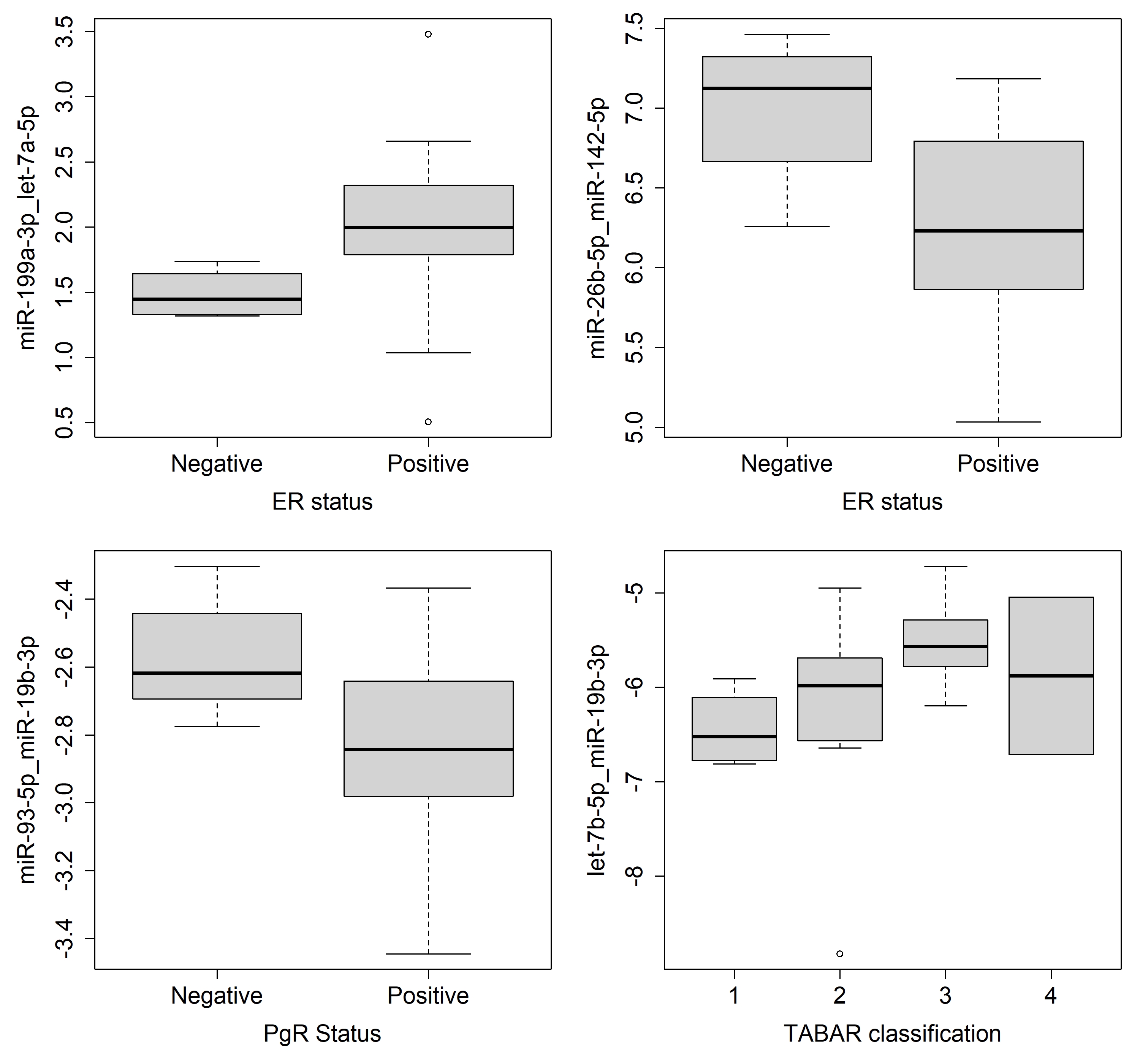


**Figure S2.** Expression values of miRNA-ratios associated with clinicopathological characteristics of BC cases.


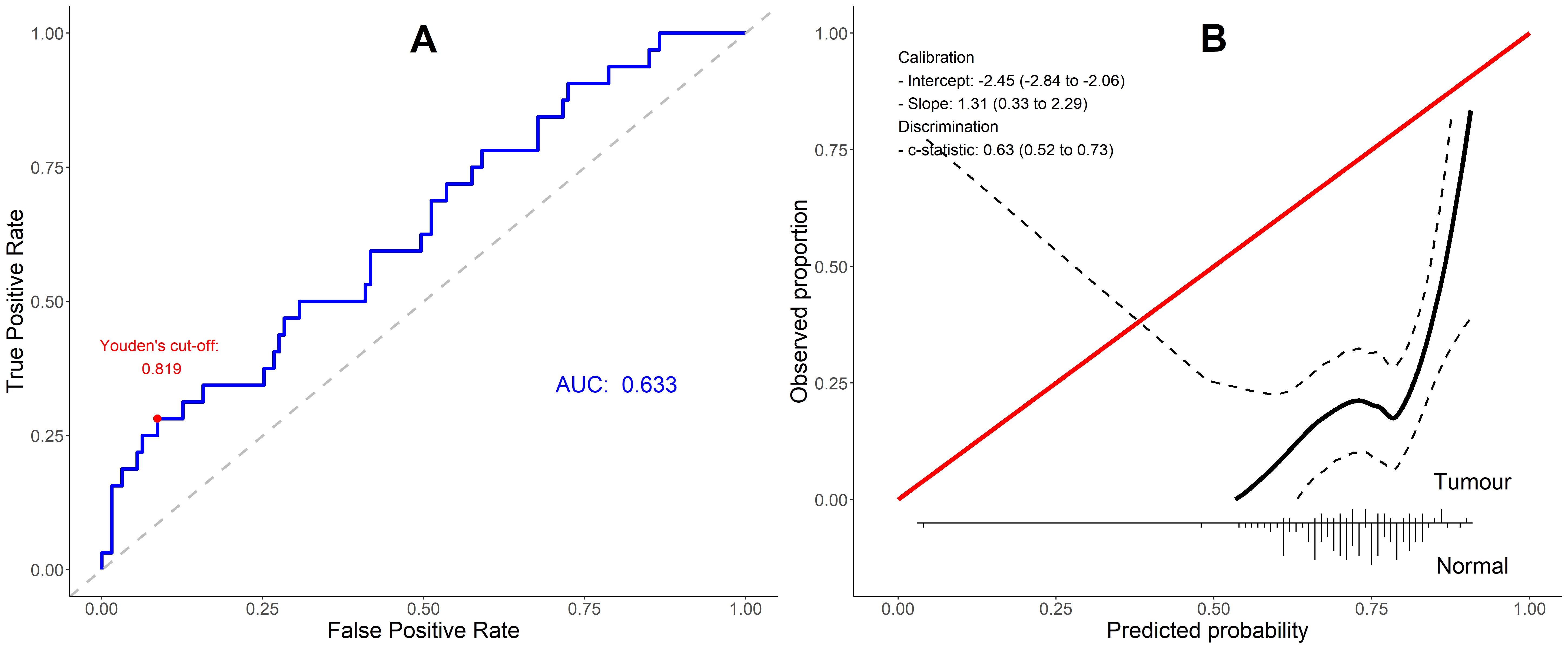


**Figure S3.** ROC AUC (A) and calibration plot (B) of the discovery set model on all predictors applied to the validation set. Within the ROC curve, the Youden’s cut-off and AUC, and within the calibration plot, the intercept and slope of the calibration curve and the c statistic are reported.


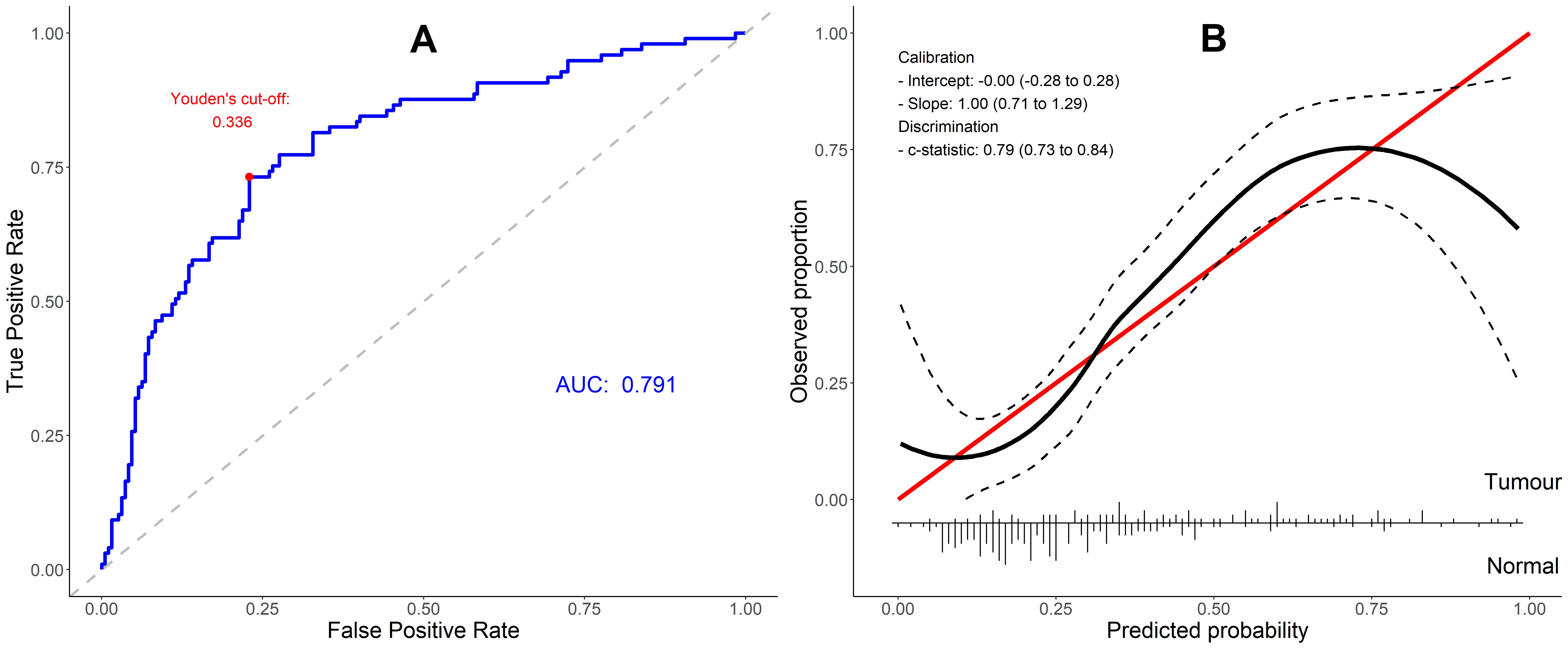


**Figure S4.** ROC AUC (A) and calibration plot (B) of the IECV model on the most generalizable predictors. Within the ROC curve, the Youden’s cut-off and AUC, and within the calibration plot, the intercept and slope of the calibration curve and the c statistic are reported.


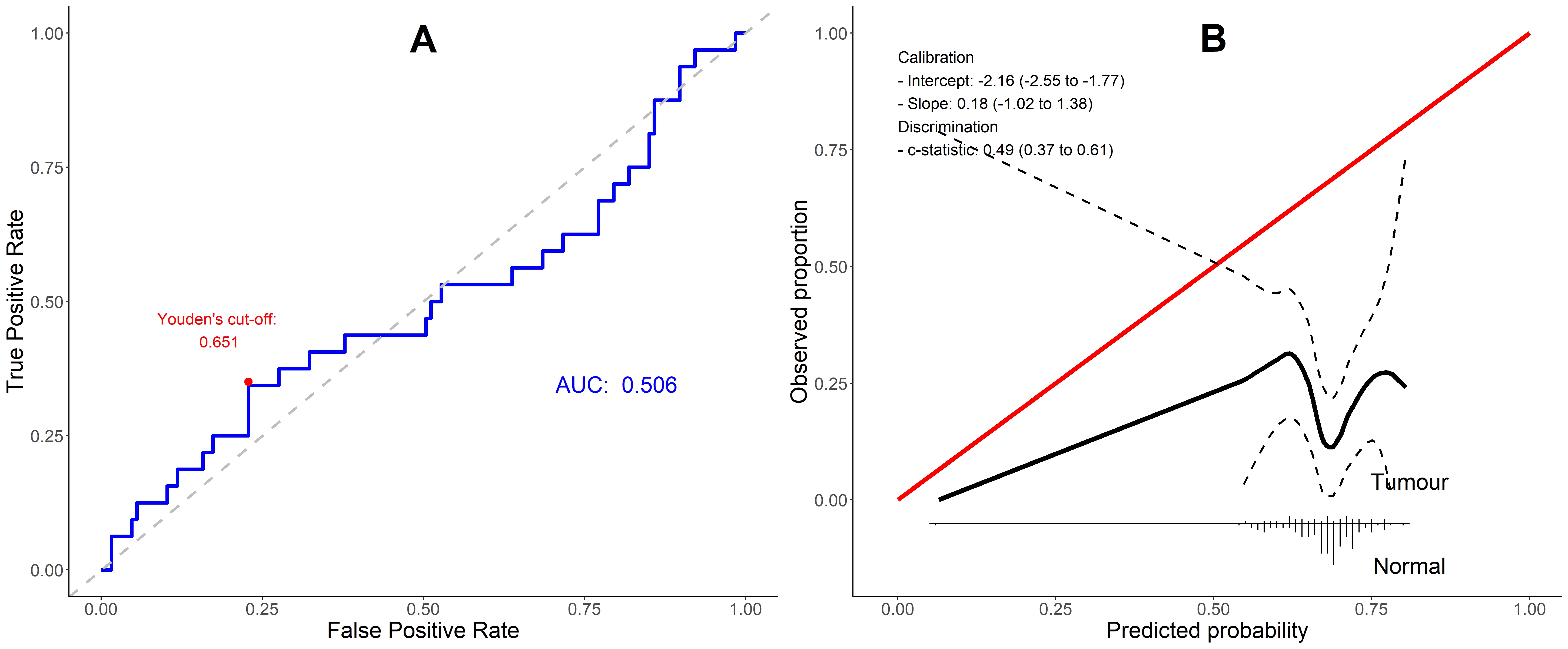


**Figure S5.** ROC AUC (A) and calibration plot (B) of the discovery set model on miRNA ratios applied to the validation set. Within the ROC curve, the Youden’s cut-off and AUC, and within the calibration plot, the intercept and slope of the calibration curve and the c statistic are reported.


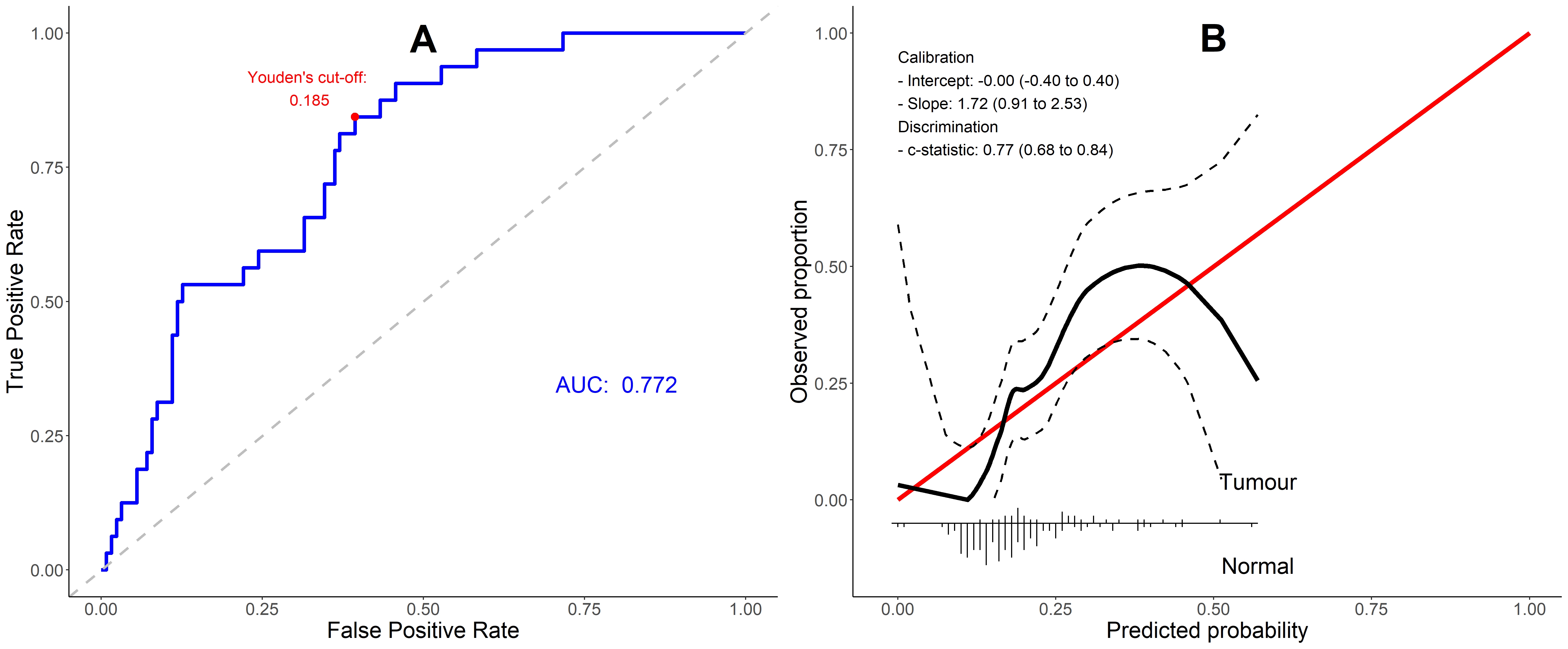


**Figure S6.** ROC AUC (A) and calibration plot (B) of the ridge regression model (model recalibration) on miRNA ratios. Within the ROC curve, the Youden’s cut-off and AUC, and within the calibration plot, the intercept and slope of the calibration curve and the c statistic are reported.
