# Supplementary material for: Confirmation of previously identified plasma microRNA ratios for breast cancer detection in a nested case‐control study within a screening setting
Source: Clin Transl Med. 2024 Nov 15;14(11):e70068. doi: 10.1002/ctm2.70068 (PMC11567874; doi:10.1002/ctm2.70068)
Supplement: Supplementary file 3 — Supporting Information [file CTM2-14-e70068-s005.docx]

Table S1. Histological and molecular subtype characteristics of invasive and in situ breast cancer cases of the validation set.

| **Invasive (n = 31)** | | | **In situ (n = 1)** | | |
| --- | --- | --- | --- | --- | --- |
|  | N | % |  | N | % |
| **Histotype** | | | **Histotype** | | |
| Ductal NOS | 21 | 75.00 | Ductal NOS | 0 | 0 |
| Lobular | 4 | 14.29 | Solid | 1 | 100 |
| Tubular | 0 | 0 | Micropapillary | 0 | 0 |
| Other | 3 | 10.71 | Papillary | 0 | 0 |
| Missing | 3 |  | Other | 0 | 0 |
| **Grade** | | | **Grade** | | |
| I | 3 | 10.34 | I | 0 | 0 |
| II | 16 | 55.17 | II | 1 | 100 |
| III | 10 | 34.48 | III | 0 | 0 |
| Missing | 2 |  | **Tumour size (mm)** | | |
| **pT** | | | 1–10 | 0 | 0 |
| 1a-1b-1mic | 7 | 25.00 | 11–20 | 1 | 100 |
| 1c | 12 | 42.86 | 21 + | 0 | 0 |
| 2 + | 9 | 32.14 |  |  |  |
| Missing | 3 |  |  |  |  |
| **Tumour size (mm)** | | |  |  |  |
| 1–10 | 7 | 25.93 |  |  |  |
| 11–20 | 12 | 44.44 |  |  |  |
| 21 + | 8 | 29.63 |  |  |  |
| Missing | 4 |  |  |  |  |
| **Stage** | | |  |  |  |
| IA | 14 | 50.00 |  |  |  |
| IIA | 6 | 21.43 |  |  |  |
| IIB | 4 | 14.29 |  |  |  |
| IIIA | 3 | 10.71 |  |  |  |
| IIIC | 1 | 3.57 |  |  |  |
| IV | 0 | 0 |  |  |  |
| Missing | 3 |  |  |  |  |
| **Molecular subtypes** | | |  |  |  |
| **ER** | | |  |  |  |
| Negative | 4 | 14.81 |  |  |  |
| Positive (> 10%) | 23 | 85.19 |  |  |  |
| Missing or undetermined | 4 |  |  |  |  |
| **PgR** | | |  |  |  |
| Negative | 6 | 22.22 |  |  |  |
| Positive (> 10%) | 21 | 77.78 |  |  |  |
| Missing or undetermined | 4 |  |  |  |  |
| **Her2** | | |  |  |  |
| Negative | 23 | 88.46 |  |  |  |
| Positive | 3 | 11.54 |  |  |  |
| Missing or undetermined | 5 |  |  |  |  |
| **Ki-67** | | |  |  |  |
| Negative | 5 | 17.86 |  |  |  |
| Positive (> 20%) | 23 | 82.14 |  |  |  |
| Missing or undetermined | 3 |  |  |  |  |
| **Intrinsic subtype** | | |  |  |  |
| Luminal A-like | 4 | 17.39 |  |  |  |
| Luminal B-like (HER2 negative) | 15 | 65.22 |  |  |  |
| Luminal B-like (HER2 positive) | 2 | 8.70 |  |  |  |
| HER2 positive (non-luminal) | 0 | 0 |  |  |  |
| Triple negative | 2 | 8.70 |  |  |  |
| Missing | 8 |  |  |  |  |

NOS not otherwise specified, pT pathologic evaluation of tumour size, ER estrogen receptor, PgR progesterone receptor, Her2 human epidermal growth factor receptor 2.
