# Supplementary material for: Confirmation of previously identified plasma microRNA ratios for breast cancer detection in a nested case‐control study within a screening setting
Source: Clin Transl Med. 2024 Nov 15;14(11):e70068. doi: 10.1002/ctm2.70068 (PMC11567874; doi:10.1002/ctm2.70068)
Supplement: Supplementary file 4 — Supporting Information [file CTM2-14-e70068-s001.docx]

Table S2. Comparison of the 12 predictors analysed in the present case-control study between controls which underwent a biopsy due to a suspicious mammography and controls with a negative mammography.

|  | **Controls without second level investigation (n=96)** | | **Controls with second level investigation (n=31)** | |  |
| --- | --- | --- | --- | --- | --- |
|  | **N** | **%** | **N** | **%** | **P-value*** |
| **TABAR classification** | | | | | 0.754 |
| 1 | 40 | 41.67 | 10 | 32.26 |  |
| 2 | 45 | 46.88 | 18 | 58.06 |  |
| 3 | 7 | 7.29 | 2 | 6.45 |  |
| 4 or 5 | 4 | 4.17 | 1 | 3.23 |  |
| Missing | 0 |  | 0 |  |  |
| **BMI** | | | | | 0.635 |
| ≥ 30 | 18 | 18.75 | 4 | 12.90 |  |
| < 30 | 78 | 81.25 | 27 | 87.10 |  |
| Missing | 0 |  | 0 |  |  |
| **Menopausal status** | | | | | 0.537 |
| Yes | 81 | 84.38 | 24 | 77.42 |  |
| No | 15 | 15.63 | 7 | 22.58 |  |
| Missing | 0 |  | 0 |  |  |
| **miR-199a-3p_let-7a-5p** |  |  |  |  |  |
| mean ± SD | 1.61 ± 0.36 | | 1.56 ± 1.66 | | 0.017 |
| **miR-26b_miR-142-5p** |  |  |  |  |  |
| mean ± SD | 6.43 ± 0.50 | | 6.23 ± 0.69 | | 0.099 |
| **miR-101-3p_miR-19b-3p** |  |  |  |  |  |
| mean ± SD | -8.52 ± 0.48 | | -8.53 ± 0.43 | | 0.768 |
| **miR-93-5p_miR-19b-3p** |  |  |  |  |  |
| mean ± SD | -2.72 ± 0.21 | | -2.76 ± 0.24 | | 0.233 |
| **miR-21-5p_miR-23a-5p** |  |  |  |  |  |
| mean ± SD | 3.65 ± 0.53 | | 3.61 ± 0.53 | | 0.604 |
| **let-7a-5p_miR-19b-3p** |  |  |  |  |  |
| mean ± SD | -5.59 ± 0.74 | | -5.21 ± 1.62 | | 0.430 |
| **let-7a_miR-22-3p** |  |  |  |  |  |
| mean ± SD | 3.13 ± 2.70 | | 2.07 ± 4.33 | | 0.260 |
| **WCRF life-style score** |  |  |  |  |  |
| mean ± SD | 5.47 ± 1.10 | | 5.07 ± 1.41 | | 0.171 |

*Wilcoxon two-sample test performed on continuous variable and Pearson's chi-squared test on categorical variables
