# Supplementary material for: Confirmation of previously identified plasma microRNA ratios for breast cancer detection in a nested case‐control study within a screening setting
Source: Clin Transl Med. 2024 Nov 15;14(11):e70068. doi: 10.1002/ctm2.70068 (PMC11567874; doi:10.1002/ctm2.70068)
Supplement: Supplementary file 5 — Supporting Information [file CTM2-14-e70068-s006.docx]

Table S3. Univariate logistic regression results of seven miRNA-ratios identified in our study compared to six other circulating miRNA studies which included BC patients and healthy controls. For each dataset we report the GSE identification number as well as the number of BC cases (CA) and healthy controls (CO). Details on the included studies (including the citations), data processing and statistical methods are found in supplementary materials.

| **miRNA Ratio** | **Validation ANDROMEDA, (CA/CO: 32/127)^‡^** | **GSE22981 (CA/CO: 20/20)^§^** | **GSE118782 (CA/CO: 30/10)^§^** | **GSE41526  (CA/CO: 20/20)^§^** | **GSE41922  (CA/CO: 32/22)^‡^** | **GSE73002 (CA/CO: 1277/2683)^§^** | **GSE113486 (CA/CO: 40/52)^§^** |
| --- | --- | --- | --- | --- | --- | --- | --- |
|  | OR (95% CI);  p-value | OR (95% CI);  p-value | OR (95% CI);  p-value | OR (95% CI); p-value | OR (95% CI);  p-value | OR (95% CI);  p-value | OR (95% CI);  p-value |
| miR-199a-3p/let-7a-5p | 2.96 (1.23-7.57); **0.019** | 0.76 (0.53-1.01); 0.089 | 2.70 (1.11-10.59); 0.082 | 0.27 (0.06-1.04); 0.074 | 2.99 (1.64-6.34); **0.001** | 1.40 (1.28-1.53); **<0.001** | 1.11 (0.99-1.24); 0.073 |
| miR-26b-5p/miR-142-5p | 1.26 (0.64-2.53); 0.504 | 0.95 (0.78-1.16); 0.624 | 1.31 (0.19-8.42); 0.770 | 0.85 (0.72-0.97); **0.032** | 0.15 (0.04-0.38); **<0.001** | 0.93 (0.84-1.02); 0.139 | 0.81 (0.70-0.93); **0.003** |
| let-7b-5p/miR-19b-3p | 0.52 (0.30-0.87); **0.016** | 1.08 (0.85-1.39); 0.521 | 0.68 (0.42-0.95); 0.053 | 0.41 (0.15-0.88); **0.049** | NA* | 1.23 (1.14-1.32); **<0.001** | 1.08 (0.96-1.22); 0.200 |
| miR-101-3p/miR-19b-3p | 1.18 (0.56-2.66); 0.671 | 1.14 (0.93-1.44); 0.224 | 1.20 (0.80-1.89); 0.390 | 1.59 (1.06-2.69); **0.047** | NA* | 1.29 (1.20-1.39); **<0.001** | 0.84 (0.73-0.96); **0.011** |
| miR-93-5p/miR-19b-3p | 0.39 (0.07-2.19); 0.286 | 0.99 (0.76-1.29); 0.929 | 0.45 (0.20-0.86); **0.032** | 0.32 (0.10-0.80); **0.038** | NA* | 1.60 (1.46-1.76); **<0.001** | 1.12 (0.99-1.28); 0.069 |
| let-7a-5p/miR-22-3p | 1.17 (1.03-1.33); **0.016** | 1.18 (0.90-1.64); 0.263 | 0.96 (0.62-1.58); 0.870 | 0.63 (0.17-1.71); 0.402 | 1.06 (0.59-1.91); 0.856 | 1.55 (1.47-1.64); **<0.001** | 0.89 (0.78-1.01); 0.089 |
| miR-21-5p/mir-23a-3p | 1.98 (1.03-3.92); **0.044** | 1.21 (0.48-4.04); 0.679 | 2.28 (1.23-13.98); 0.137 | 3.64 (0.14-131.97); 0.447 | 0.27 (0.09-0.71); **0.013** | 1.68 (1.59-1.78); **<0.001** | 1.04 (0.90-1.21); 0.570 |

Datasets based on qRT-PCR were marked with ‡, while datasets based on next generation sequencing and microarray platforms were marked as # and §, respectively.

*miR-19b-3p was not found in GSE41922 dataset
