# Supplementary material for: Confirmation of previously identified plasma microRNA ratios for breast cancer detection in a nested case‐control study within a screening setting
Source: Clin Transl Med. 2024 Nov 15;14(11):e70068. doi: 10.1002/ctm2.70068 (PMC11567874; doi:10.1002/ctm2.70068)
Supplement: Supplementary file 6 — Supporting Information [file CTM2-14-e70068-s002.docx]

Table S4. Paired Mann Whitney U test on the 7 candidate miRNA ratios in the BC and adjacent healthy tissue TCGA miRNA expression dataset.

| **miRNA ratio** | **FC** | **log_2_FC** | **P-value** |
| --- | --- | --- | --- |
| miR-199a-3p/let-7a-5p | 0.861 | -0.215 | <0.001 |
| miR-26b-5p/miR-142-5p | 0.783 | -0.353 | <0.001 |
| let-7b-5p*/miR-19b-3p | 0.948 | -0.076 | 0.004 |
| miR-101-3p/miR-19b-3p | 0.956 | -0.064 | 0.020 |
| miR-93-5p/miR-19b-3p | 1.197 | 0.260 | <0.001 |
| let-7a-5p/miR-22-3p | 1.552 | 0.634 | 0.612 |
| miR-21-5p/miR-23a-3p | 1.719 | 0.782 | <0.001 |

*Due to the availability of miRNA expression data for let-7b-5p in the TCGA dataset, here it was not replaced by let-7a-5p. The same expression difference was anyway observed for let-7a-5p/miR-19b-3p ratio.
